# Headaches; Their Nature, Cause and Treatment

**Published:** 1880-04

**Authors:** 


*Headaches; Their Nature, Cause and Treatment.* By William Henry Day, M. D., Member R. C. P., London, Etc., Etc. Third Edition with Illustrations. Publishers: Lindsay & Blackiston, Philadelphia, 1880.

A useful manual in which the whole class of headaches is carefully and intelligently treated under special divisions, the nature, origin and treatment of each being clearly described. Headaches in childhood and youth are minutely noticed, and excellent directions given for the observance of parents and teachers in their management of children, all of which greatly add to the value of this treatise. The peculiarities of the cerebral circulation, and the relationship existing between the nerves and blood-vessels are clearly defined in order that nervous pain may be fairly comprehended. Accompanying the treatment are many useful formulæ, which, with the views advanced, are the results of notes and observations recorded by the author during a professional experience of many years.

This work cannot fail to impart useful information and prove an important adjunct to general medical practice.

